# Supplementary figures and images for: Patterns of gene evolution following duplications and speciations in vertebrates
Source: PeerJ. 2020 Mar 31;8:e8813. doi: 10.7717/peerj.8813 (PMC7120047; doi:10.7717/peerj.8813)

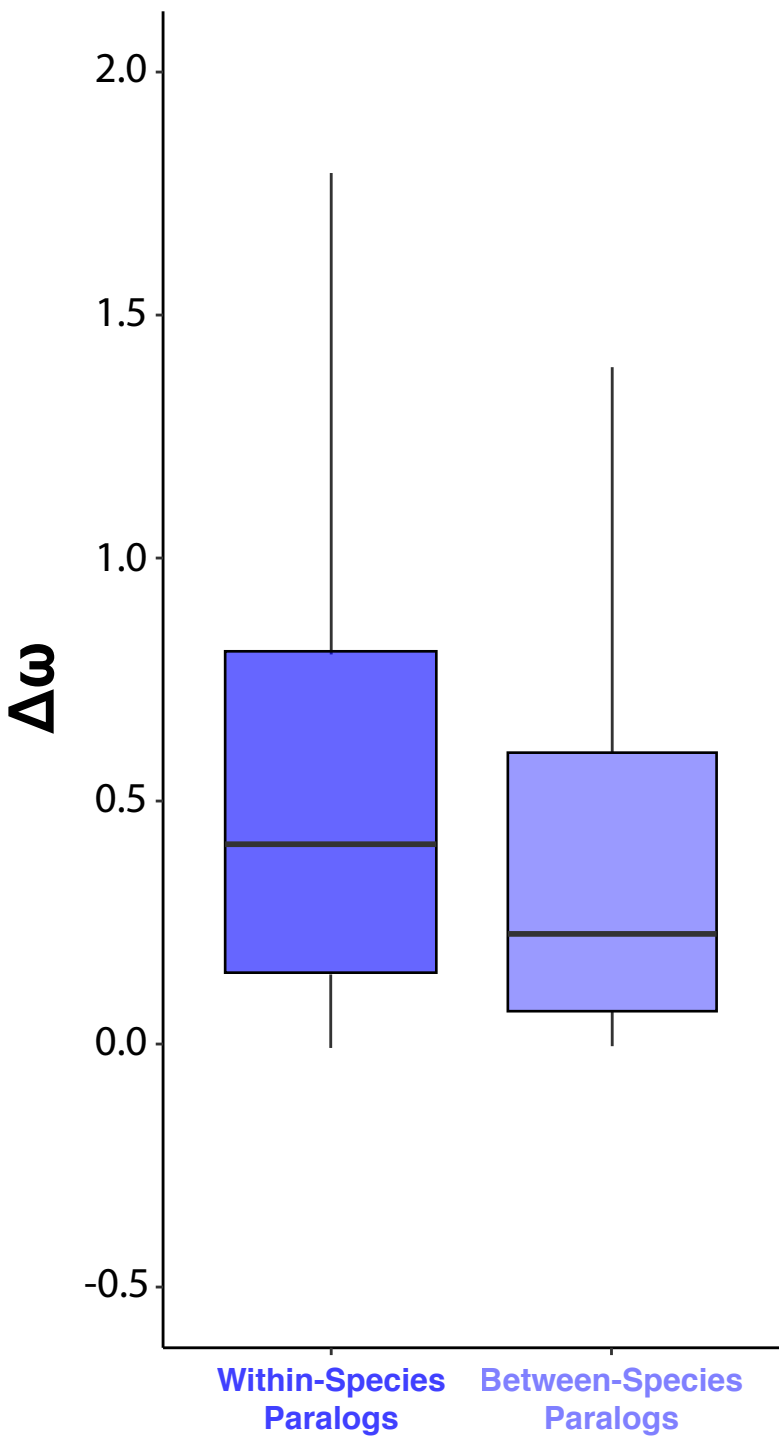

Supplement: Supplemental Information 3 — Boxplots of Δω for both within-species and between-species paralogs. Lineages leading to within-species paralogs have significantly (p < 0.001) higher Δω than lineages leading to between-species paralogs. [file peerj-08-8813-s003.pdf]

**3**

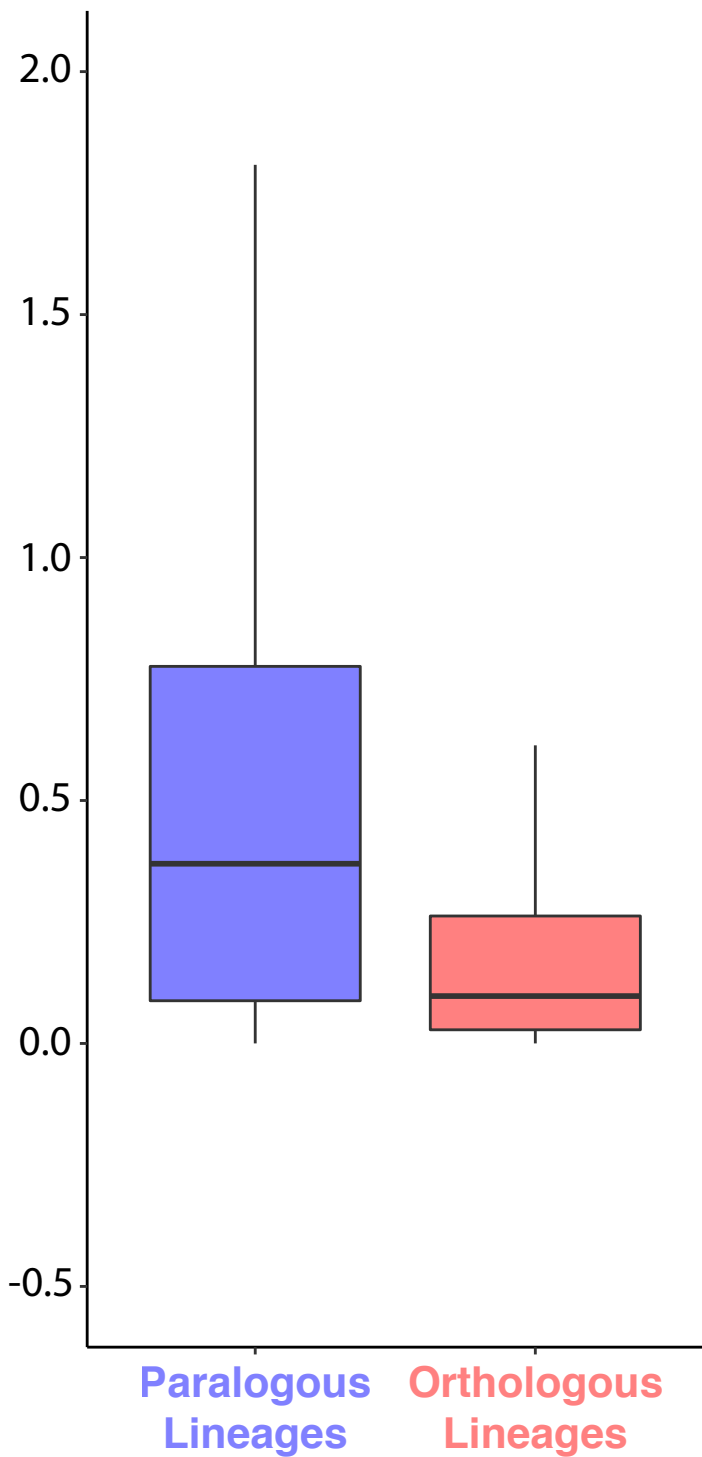

Supplement: Supplemental Information 4 — Boxplots of ω for orthologous and paralogous lineages. Paralogous lineages have significantly (p<0.001) higher ω than orthologous lineages. [file peerj-08-8813-s004.pdf]

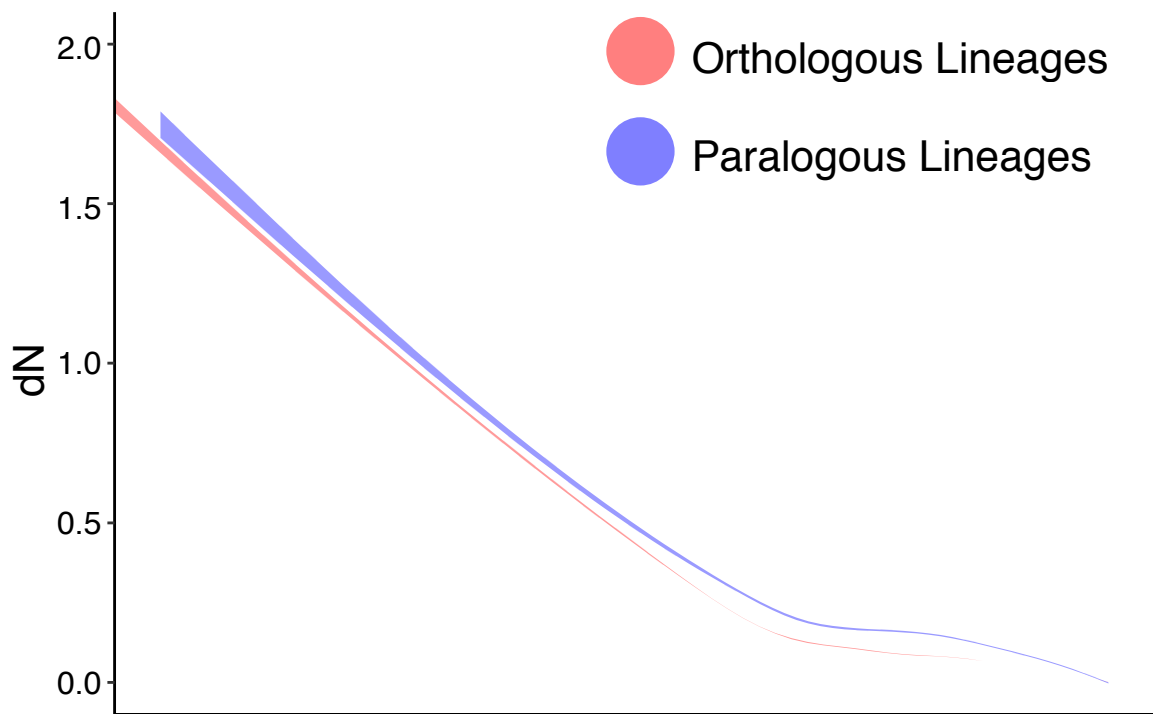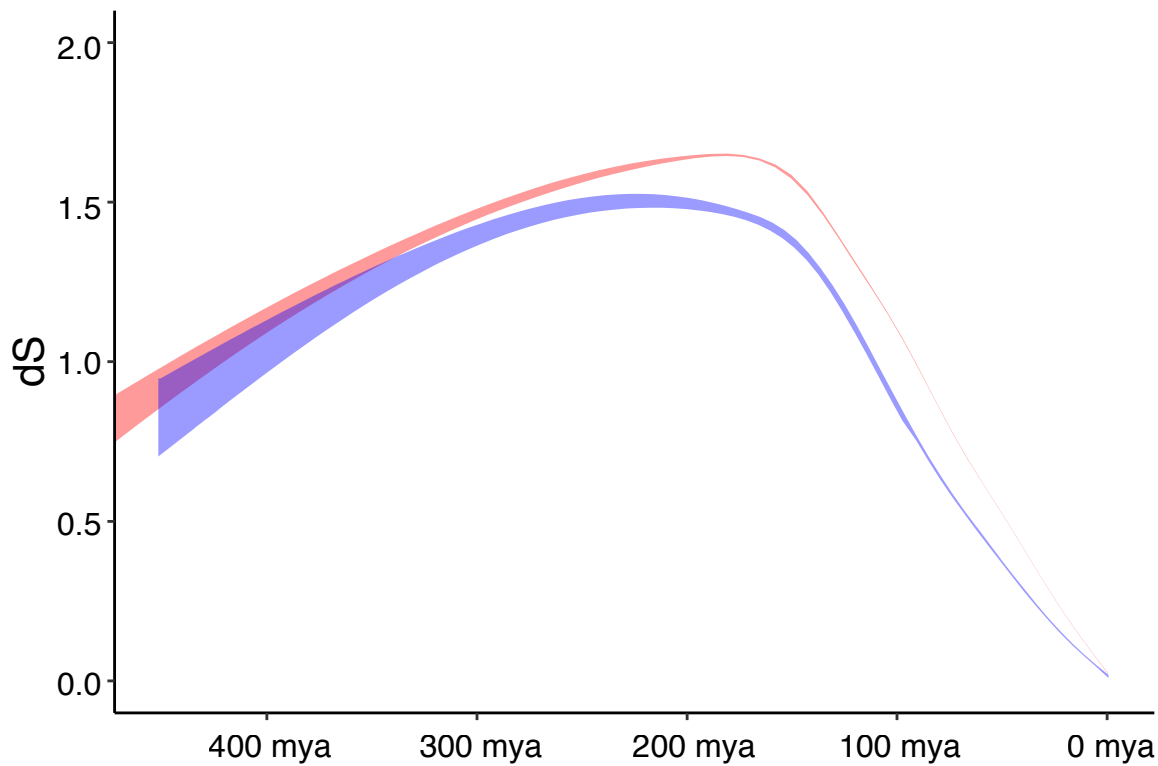

Supplement: Supplemental Information 5 — General additive model 95% confidence intervals of dN and dS over time, for orthologous and paralogous lineages. [file peerj-08-8813-s005.pdf]
